# A Clustered Adaptive Exposure Time Selection Methodology for HDR Structured Light 3D Reconstruction

**DOI:** 10.3390/s25154786

**Published:** 2025-08-03

**Authors:** Zhuang Li, Rui Ma, Shuyu Duan

**Affiliations:** 1 Tsinghua Shenzhen International Graduate School, Tsinghua University, Shenzhen 518055, China; liz22@mails.tsinghua.edu.cn; 2 School of Electronic Engineering, Guangxi University of Science and Technology, Liuzhou 545616, China; 2013026@gxust.edu.cn

**Keywords:** exposure fusion selection, high dynamic range, fringe projection profilometry

## Abstract

Fringe projection profilometry (FPP) has been widely applied in industrial 3D measurement due to its high precision and non-contact advantages. However, FPP often encounters measurement problems with high-dynamic-range objects, consequently impacting phase computation. In this paper, an adaptive exposure time selection method is proposed to calculate the optimal number of exposures and exposure time by using an improved clustering method to divide the region with different reflection degrees. Meanwhile, the phase order sharing strategy is adopted in the phase unwrapping stage, and the same set of complementary Gray code patterns is used to calculate the phase orders under different exposure times. The experimental results demonstrate that the measurement error of the method described in this paper was reduced by 25.4% under almost the same exposure times.

## 1. Introduction

Optical measurement in industrial visual inspection utilizes light-based technologies to capture, analyze, and quantify the visual features of products or components, such as dimensions, surface defects, and color consistency, thereby enabling non-contact, high-precision, and efficient quality control in manufacturing processes [1,2,3,4]. Fringe projection profilometry (FPP) is a commonly used method for 3D measurement [5,6]. Compared with other methods such as stereo vision [7,8] and line laser scanning [9,10], FPP has the advantages of high accuracy and fast speed [11]. Its main technical process includes coded projection [12], camera acquisition, system calibration [13], phase solving, and point cloud reconstruction [14,15,16,17,18,19]. In pursuit of enhancing measurement precision and operational efficiency, researchers have dedicated their efforts to optimizing different aspects, including projection devices [20,21,22,23], calibration methods [24,25,26], phase recovering algorithms [15,16,27,28], and deep learning [5,28,29,30]. However, when performing 3D measurements of objects in real production, the measurement effectiveness of structured light systems is often affected by the optical properties of the surface of the object being measured [31]. In general, high-dynamic-range (HDR) objects are those with a wide range of surface reflectance variations, such as rusty, oily, or shiny surfaces, which result in a dynamic range that exceeds the ability of traditional low-dynamic-range sensors to capture images [32,33]. In industrial inspection, they refer to workpieces with surfaces that are under-modulated and have oversaturated pixels in the captured image due to differences in the material.

Adjusting the exposure time is a common method used to cope with the 3D measurement of HDR scenes, and researchers have proposed a series of multiple exposure-based techniques to measure high-dynamic-range objects [34,35,36,37]. Feng et al. [23] proposed a fringe projection method that can inspect scenes with a large range of reflectivity variation. Rao et al. [24] carry out high-dynamic-range 3D shape determination based on automatic exposure selection. The images collected at different exposure times are arranged in order from brightest to darkest. The image is then traversed pixel by pixel to select the brightest, yet unsaturated, pixels, generating a new fused image for subsequent absolute phase recovery. The fused image has a good striation system for both the bright and dark areas of the streak image, enabling the method to be used for effective measurements of different reflective surfaces. However, the selection of the method’s multiple exposure parameters is based on experience rather than a specific calculation method. Additionally, scenes with too large a dynamic range need multiple exposures, the measurement speed is low, the number of projected coded images in the paper is high, and the measurement efficiency is low.

Research has also been conducted regarding the selection of exposure time, with some researchers developing an exposure time selection method based on pixel analysis [38,39,40,41]. Lin et al. [29] present a new adaptive digital fringe projection technique that avoids image saturation and has a high signal-to-noise ratio (SNR) in the three-dimensional (3D) shape measurement of objects that have a large range of reflectivity variation across their surfaces. Jiang et al. [30] proposed 180-degree phase-shifted (or inverted) fringe patterns to complement regular fringe patterns. This method measures the reflectance characteristics of the target surface pixel by pixel and then constructs the optimal exposure sequence. The core process involves acquiring image sequences under different projection intensities, establishing a pixel intensity–projection intensity response model, and determining the optimal exposure parameters by calculating the reflectance gradient. However, the pixel-by-pixel nature of this method leads to a significant increase in the complexity of the algorithm, which makes it difficult to meet real-time demands and does not take into account how the number of exposure groups should be divided.

Existing exposure assessment metrics are mainly designed based on the principles of gradient maximization [42] or information entropy maximization [43], which are theoretically based on the fact that conventional vision algorithms rely on gradient or entropy features for target recognition. In structured light 3D measurement, however, it is the integrity of the phase encoding information that is the core factor in determining the 3D reconstruction accuracy. For this reason, Liu et al. [44] proposed and experimentally verified the effectiveness of a phase information evaluation index based on intensity modulation, which assumes that the quality of phase encoding is positively correlated with the intensity of stripe modulation [45]. However, this scheme is only applicable to short exposure control in low illumination scenes. When facing the wide reflectance distribution (0.05–0.95) of industrial parts, the long exposure strategy will lead to overexposure of the high-dynamic-range region, which seriously affects the phase decoding accuracy.

In this paper, we propose incorporating complementary Gray codes into the multi-exposure fusion method to improve measurement efficiency. Generally, the multi-frequency method is the unwrapping algorithm used in multi-exposure fusion, which leads to a larger number of projected images and lower measurement efficiency when applied to multi-exposure techniques. On the other hand, complementary Gray codes are binary encodings used solely for unwrapping and possess advantages such as high reliability and robustness to noise. More importantly, they only require the projection of one set, thereby improving the efficiency of 3D measurement. This paper discusses the principles and methods for implementing multi-exposure fusion combined with complementary Gray codes. Experimental results demonstrate the effectiveness and speed of this method.

## 2. Methodology

Structured light 3D measurement generally consists of four parts: encoded projection, camera acquisition, phase unwrapping, and point cloud reconstruction. The method proposed in this paper focuses on improving the two parts of camera acquisition and phase unwrapping. The adaptive exposure fusion method determines the optimal number of exposures and exposure time required in HDR scenes through clustering. The phase level sharing method based on complementary Golay code refers to using the same set of Golay code patterns for phase unwrapping of phase-shift images captured under different exposure times, thereby reducing the number of projection images in HDR scenes. The specific contents of these two methods are introduced below.

### 2.1. Adaptive Exposure Time Selection Method

**Figure 1:**
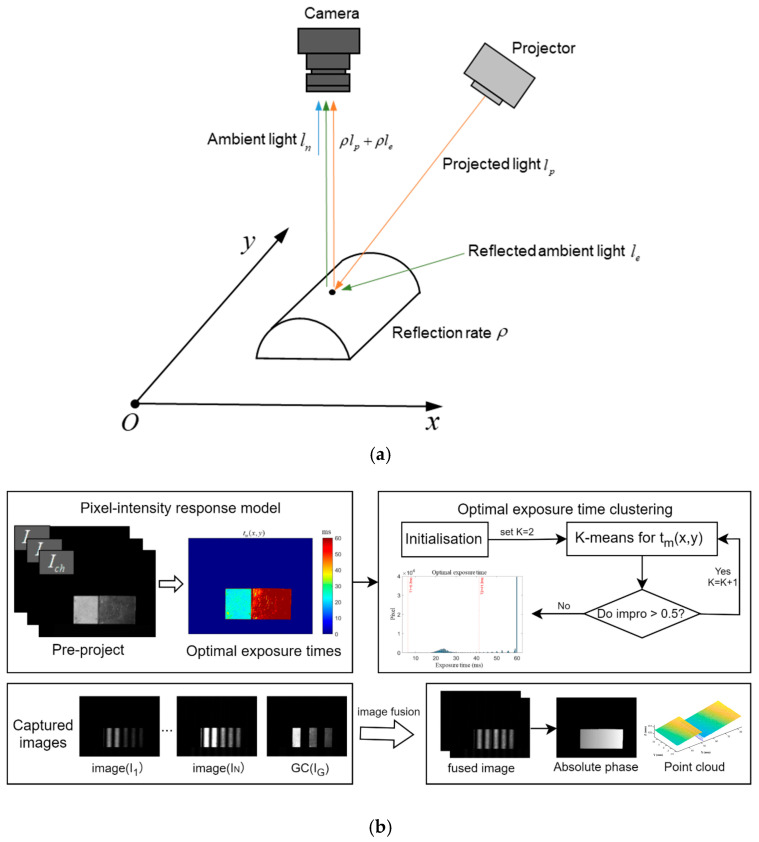
The pipeline of our adaptive exposure time selection method. (**a**) Pixel-projected intensity response model. (**b**) The clustered adaptive exposure time selection flowchart.

The grey scale of an image captured by the camera is affected by the external light, camera gain, and reflectivity of the captured object and is described by the following equation, Equation (1):

(1) $$I_{c}(x,y;t)=t\alpha (\rho (x,y)l_{p}(x,y)+\rho (x,y)l_{e}(x,y))+l_{n}(x,y)$$

where $I_{c}(x,y;t)$ is the brightness of the image obtained by the camera sensor at the pixel coordinates $\left(x,y\right)$ with the exposure time, t is the exposure time, α is the camera’s scale factor for converting the incoming light intensity into grey values, ρ(x,y) is the reflectivity of the illuminated object, $l_{p}\left(x,y\right)$ is the intensity of the projector’s projection at the pixel coordinates, and $l_{e}\left(x,y\right)$ is the intensity of the ambient light on the surface of the object. $l_{n}\left(x,y\right)$ includes the camera’s own noise and the ambient light that enters directly into the lens. Figure 1a shows our pixel-projection intensity mapping model.

The projected luminance is linearly related to the image greyness as follows:

(2) $$\left\{\begin{array}{l} I_{c}\left(x,y;t\right)=K\left(x,y\right)l_{p}\left(x,y\right)+B\left(x,y\right)\\ K\left(x,y\right)=t\alpha \rho \left(x,y\right)\\ B\left(x,y\right)=t\alpha \rho \left(x,y\right)l_{e}\left(x,y\right)+l_{n}\left(x,y\right) \end{array}\right.$$

If $l_{p}\left(x,y\right)$ is known, tα is constant after a fixed exposure time, so that two different pictures of known brightness are projected through a projector, and the reflectance $\rho \left(x,y\right)$ of an object at each pixel point within the imaging range of the camera can be calculated by capturing the two pictures.

Assume that the projector pre-projects a uniform grey scale image with an intensity of $l_{pl}$ = 51 and $l_{ph}$ = 255 and captures image values of $I_{cl}\left(x,y;t\right)$ and $I_{ch}\left(x,y;t\right)$. If there are no oversaturated pixels in the current $I_{ch}\left(x,y;t\right)$, the reflectance of the object at this time is calculated using Equations (3) and (4):

(3) $$K\left(x,y\right)=\left\{\begin{array}{l} \frac{I_{ch}\left(x,y;t\right)-I_{cl}\left(x,y;t\right)}{l_{ph}\left(x,y;t\right)-l_{pl}\left(x,y;t\right)}\;I_{ch}\left(x,y\right)<255\\ \frac{I_{cl}\left(x,y;t\right)-B\left(x,y\right)}{l_{pl}\left(x,y\right)}\;I_{ch}\left(x,y\right)=255\;\mathrm{and}\;I_{cl}\left(x,y\right)<255 \end{array}\right.$$

(4) $$B(x,y)=I_{c0}\left(x,y;t\right)=t\alpha \rho \left(x,y\right)l_{p}\left(x,y\right)+l_{e}(x,y)$$

In this case, if there are saturated pixels in $I_{ch}\left(x,y;t\right)$, it is necessary to set the projected brightness to 0, set tα to a constant value, and acquire the corresponding image $I_{c0}$, when $I_{c0}\left(x,y\right)=B\left(x,y\right)$.

If the light intensity of the captured image is too dark, it will make the overall signal-to-noise ratio low, and the phase information will be easily affected by the camera noise and ambient light. If the light intensity is too strong, there will be a loss of phase information in the grey saturated region, so the ideal pixel grey value $I_{th}$ is set to be slightly below the upper limit of the camera’s grey level (the threshold for setting the maximum unsaturated value is <255, which is generally set to 250, it is necessary to leave some redundancy to reduce the error), and then the optimal exposure time can be expressed as follows:

(5) $$t_{m}\left(x,y\right)=\left\{\begin{array}{l} \frac{I_{th}-B\left(x,y\right)}{I_{ch}-B\left(x,y\right)}\frac{l_{p}\left(x,y\right)}{l_{ph}\left(x,y\right)}t_{0},\;I_{ch}\left(x,y\right)<255\\ \frac{I_{th}-B\left(x,y\right)}{I_{cl}-B\left(x,y\right)}\frac{l_{p}\left(x,y\right)}{l_{pl}\left(x,y\right)}t_{0},\;I_{ch}\left(x,y\right)=255\;\mathrm{and}\;I_{cl}\left(x,y\right)<255 \end{array}\right.$$

Ideal exposure time is largely determined by the degree of surface reflectance of the object, which in turn is largely dependent on the material of the object. For objects with a high degree of surface reflectance, the reflectance distribution will have several peaks depending on the material composition. Therefore, we can use some methods to automatically determine the top K exposure times that account for the largest percentage of the exposure time $t_{m}(x,y)$ represented, so that the exposure time sequence can be adaptively selected according to the scene.

Figure 1b illustrates the flowchart of the adaptive exposure time calculation. According to Equation (5), the optimal exposure time for each pixel position can be calculated from the results of uniform grey-scale images with pre-projected intensities $l_{pl}$, $l_{ph}$, and $l_{c0}$. By analyzing the image intensity values of the object under test, we can find a set of representative exposure time sequences. The K-means clustering algorithm is a classical and widely used automatic classification clustering method that is an unsupervised learning algorithm and can classify data into K classes based on the similarity and the distance metric. This paper proposes a strategy for adaptive exposure time selection based on the principle of K-means clustering. The strategy can divide the one-dimensional array $t_{m}=\left\{t_{m}(x_{1},y_{1}),t_{m}(x_{1},y_{1}),\ldots ,t_{m}(x_{wh},y_{wh})\right\}$ consisting of exposure values $t_{m}(x,y)$ into K groups of $C=\left\{C_{1},C_{2},C_{3},\ldots ,C_{k}\right\}$ classes. The adaptive exposure time strategy is chosen to reduce the saturated area in the image corresponding to any pixel (i, j); the light intensity of the captured image at that pixel position will be saturated if the corresponding exposure time $t_{m}\left(x_{i},y_{i}\right)$ is exceeded. Therefore, the minimum exposure value $t_{ci}$ of each group is chosen as the typical value of the group, instead of the group average value in the conventional clustering algorithm. The specific adaptive exposure time strategy is as follows:

Step 1: Pre-projection image acquisition. A uniform grey-scale map with projection intensities of $l_{pl}$ = 51 and $l_{ph}$ = 255 is projected onto the surface of the object under test, the corresponding reflected light field images $I_{cl}$ and $I_{ch}$ are captured by the camera, and the ambient light image $I_{c0}$ is captured in the unprojected state.

Step 2: Calculation of the ideal pixel-by-pixel exposure time $t_{m}(x,y)$. The optimal pixel-by-pixel exposure time can be calculated according to Equation (5), which serves as the sample set for subsequent cluster analysis.

Step 3: Calculation of the optimal exposure time series based on the improved K-means principle.

(1) Set the maximum number of acceptable clustering clusters $K_{\max}$, the maximum number of iterations iter, the minimum exposure time $t_{\min}$, the maximum exposure time $t_{\max}$, and the improvement threshold $impro_{th}$.
(2) An initial cluster center is randomly selected for each cluster, the sample set is assigned to the nearest neighboring cluster according to the minimum distance principle, and the cluster centers are updated using the sample means for each cluster center.
(3) Repeat step (2) until the clustering center no longer changes or the maximum number of iterations is reached.
(4) Calculate and save the sum of the current distances from each intra-cluster point to the cluster center $Dis_{k}$ (when the number of clusters k > 2), and calculate the current degree of improvement $impro=(Dis_{k}-Dis_{k-1})/Dis_{k-1}$. If $impro>impro_{th}$ and $k<K_{\max}$, add 1 to the current number of clusters k and repeat step (2); otherwise, output the final number of clusters k and the minimum value of each intra-cluster point $t_{ci}(i=1,2,\ldots ,k)$.

It is worth noting that before the actual clustering operation, the ideal exposure time $t_{m}(x,y)$ for the pixel-by-pixel calculation is truncated up and down so that the maximum value of $t_{m}(x,y)$ is $t_{\max}$ and the minimum value is $t_{\min}$, where $t_{\max}$ and $t_{\min}$ are determined according to the demand during the actual measurement. According to experience, $t_{\min}\geq 6\;\mathrm{ms}$, $t_{\max}$ < 60 ms, $impro_{th}=0.5$.

After the determination of the optimal number of exposure groups and times by the adaptive exposure time selection method, multiple image sequences under the corresponding exposure times are acquired and then fused to generate high-dynamic-range images with uniform light intensity distribution and a high image signal-to-noise ratio. The multi-exposure fusion algorithm used in this paper is similar to the multi-polarization method [46], which is based on projecting and acquiring multiple sets of phase-shifting images with different camera exposure times and then selecting the maximum unsaturated channel for each pixel from the multiple sets of phase-shifting images and ensuring that each pixel of the same frequency streak image also uses the same channel to generate the fused image, so as to reduce the number of saturated points of the image to restore the saturated region phase and to ensure that the dark region greyness is not weakening. The specific formula is as follows:

(6) $$p_{fs}=\arg\max_{n}\{I_{p_{t},n}\left(x,y\right)\;for\;n=1,2,\ldots ,N\}$$

(7) $$I_{f}(x,y)=I_{p_{fs},n}(x,y)$$

where $p_{t}$ is the maximum unsaturated exposure time channel under different exposure times in the same step stripe pattern. $p_{fs,n}$ is the exposure group sequence number corresponding to a position in the *N*-th step of the phase-shifting sequence when the maximum unsaturated luminance is obtained, also known as the fusion decision map. After the fused image $I_{f}$ is obtained, the absolute phase of each position can be solved, and the 3D point cloud information can be calculated by substituting the phase with the system calibration parameters.

### 2.2. Phase Order Sharing Method

The projection strategy in the experiments is the N-step phase-shifting method. This method is not affected by violent changes or discontinuities on the surface of the object to be measured, and it has been widely applied in fringe projection profilometry (FPP) systems [47]. The gray-value map captured by the camera can be expressed as

(8) $$I_{n}(x,y)=A(x,y)+B(x,y)\cos[\phi (x,y)+2\pi n/N]$$

where n is the phase-shifting index, the value of N in the experiments is 6, (*x*, *y*) denotes the pixel coordinates of the camera, A and B denote the average intensity modulation, respectively, and ϕ is the phase value we need to solve. The average intensity A, intensity modulation B, and phase value ϕ can be calculated by the following equations:

(9) $$A(x,y)=\frac{1}{N}\sum_{n=1}^{N}I_{n}(x,y)$$

(10) $$B(x,y)=\frac{2}{N}\sqrt{{\left(\sum_{n=1}^{N}I_{n}\sin\left(2\pi n/N\right)\right)}^{2}+{\left(\sum_{n=1}^{N}I_{n}\cos\left(2\pi n/N\right)\right)}^{2}}$$

(11) $$\phi \left(x,y\right)=\tan^{-1}\frac{\sum_{n=1}^{N}I_{n}\sin\left(2\pi n/N\right)}{\sum_{n=1}^{N}I_{n}\cos\left(2\pi n/N\right)}$$

Since the inverse tangent function is used in the calculation, the value domain solved by the phase-shifting method alone is in (−π,π], showing a truncated distribution. If we want to extend to the whole space, we need to align the phase expansion to obtain the absolute phase. We need to uniquely determine the phase order solved at each pixel position and recover the absolute phase accordingly, which is a process called phase unwrapping or absolute phase expansion.

In this paper, a 6-step phase-shifting is mainly used to calculate the wrapped phase, and then the phase orders are calculated by the complementary Gray code method. However, in high-dynamic-range scenes where multiple exposure image fusion is required to be used, with the increase in the number of exposure groups, each group needs to project a Gray code pattern for phase unwrapping, which brings the problem of image redundancy.

**Figure 2:**
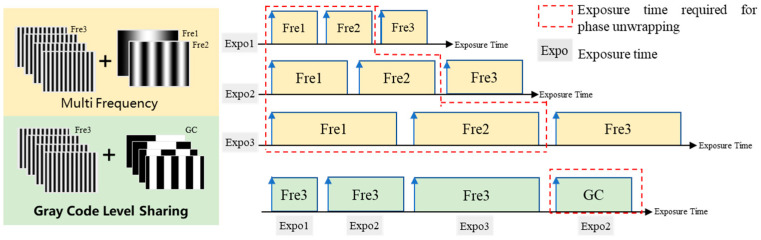
Comparison of different phase unwrapping strategies.

In contrast, the complementary-Gray-code-based phase order sharing method uses a set of complementary Gray code images of a good exposure case to compute the phase orders, which can be applied to phase unwrapping under different exposure times. This method takes advantage of the fact that the phase orders are for the same static object and the same phase expansion method. Different exposure times are basically the same, so the number of images projected in the actual high-dynamic-range measurements can be greatly reduced and the measurement efficiency can be improved under the premise of guaranteeing the correctness of the phase order solving. Figure 2 illustrates the difference between multi-exposure with the multi-frequency method and multi-exposure with the complementary Gray code method. The area of the yellow rectangle represents the projection time for the multi-frequency method, while the area of the green rectangle represents the time required for our method. In Figure 2, the 3-frequency 4-step multi-frequency method is taken as an example and compared with our method. In the multi-frequency method, Fre1 and Fre2 belong to the low-frequency stripe patterns, which are used to gradually expand the high-frequency encapsulation phase. However, in the Gray code level sharing strategy, the GC pattern performs the same function. The figure visually shows the difference in total exposure time between the two methods under the condition that the three exposure times used are exactly the same. It can be seen that our method uses less exposure time in the phase unwrapping stage, and we will prove this in the experiment in Section 3.

The principle of the phase order sharing method refers to the complementary Gray code method [48], including binarization, decoding fringe orders $k_{1}(x,y)$ and $k_{2}(x,y)$, and phase unwrapping based on segmented phase ranges, as shown in Equation (12), thereby reducing periodic order misalignment issues.

(12) $$\Phi (x,y)=\left\{\begin{array}{ll} \phi_{f}(x,y)+2\pi k_{2}(x,y), & \phi_{f}(x,y)\leq -\pi /2\\ \phi_{f}(x,y)+2\pi k_{1}(x,y), & -\pi /2<\phi_{f}(x,y)<\pi /2\\ \phi_{f}(x,y)+2\pi [k_{2}(x,y)-1], & \phi_{f}(x,y)\geq \pi /2 \end{array}\right.$$

where $\phi_{f}(x,y)$ is the new phase-shifting image sequence obtained after multiple-exposure fusion using a high-frequency sinusoidal fringe sequence. $k_{1}(x,y)$ and $k_{2}(x,y)$ are the levels calculated from the Gray code pattern of the projection acquisition at a certain well-exposed time. The exposure time is generally chosen to be the group with the largest distribution ratio in the adaptive exposure strategy.

From the above principle, it is clear that complementary Gray code images are only used for the phase unwrapping process. Therefore, when using the multi-exposure fusion algorithm, only one set of patterns is needed to complete phase unwrapping, effectively reducing projection time.

## 3. Experiments and Validation

### 3.1. System Setup

**Figure 3:**
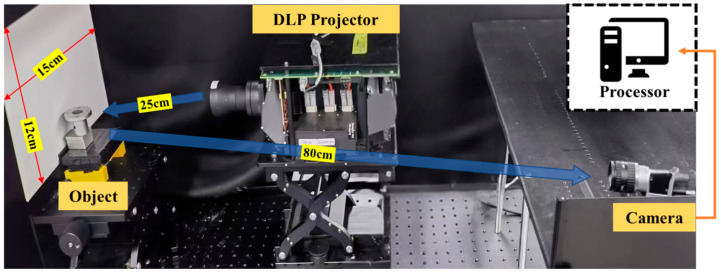
Diagram of our structured light system.

The system shown in Figure 3 consists of a DLP (digital light processing) projector (the Texas Instrument PRO6500-RGB-235 with resolution of 1920 × 1080 and a refresh rate of 247 fps @ 8 bit) and a camera (the Basler aca 2040–120 um grayscale camera, with a resolution of 2048 × 1536 and a frame rate of 119 fps). Compared to MEMS projection devices [49,50], DLP projectors offer higher precision and easier operation, making them suitable for projecting different grating patterns. The target is placed at a distance of approximately 930 mm from the camera. For the measurement tasks detailed in the following, we adopted a unidirectional structured light calibration method for system calibration [51]. We chose a six-step phase-shifting strategy with a stripe frequency of 32. The structured light patterns were projected sequentially along the vertical direction, and there were seven complementary Gray code projection patterns (five complementary-Gray-coded patterns and two pure white-and-black patterns).

### 3.2. Phase Order Sharing Experiment

In order to verify the effectiveness of the proposed complementary-Gray-code-based phase order sharing strategy, the experiments described in this section were carried out on the constructed high-dynamic-range measurement system. In these experiments, three methods, namely multi-frequency, complementary Gray code, and complementary Gray code based on phase order sharing, were chosen for phase unfolding for the convenience of control. The multi-frequency step-by-step unfolding had a sinusoidal stripe frequency ratio of 1:2:8:32, and the six-step phase-shifting method was used to calculate the parcel phase. The complementary Gray code group also used the six-step phase-shifting method. The DLP projector was set to 100ms for the projection exposure time for both the 8-bit sinusoidal stripe map and the 1-bit Gray code map, and the camera exposure time was divided into three groups of exposure times, short, medium, and long, which were 6 ms, 30 ms, and 48 ms, respectively, smaller than the projection period.

**Figure 4:**
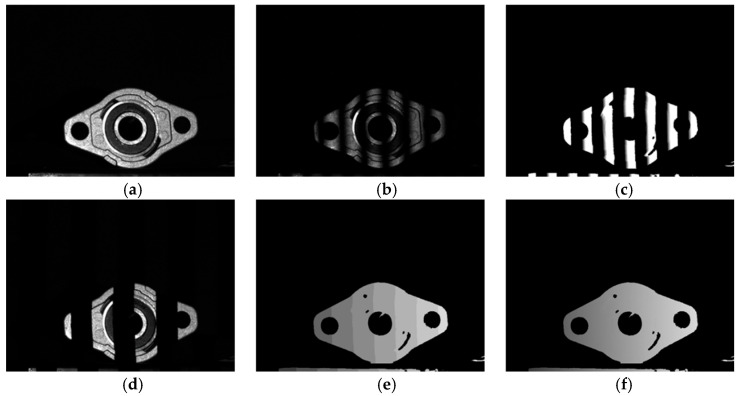
Raw image acquired with camera exposure time of 30 ms and phase resolution results. (**a**) Original object image; (**b**) phase-shifting image at the selected camera exposure time = 30 ms; (**c**) parcel phase map; (**d**) projected complementary Gray code image; (**e**) phase order image; (**f**) absolute phase map.

For this section, the outer spherical bearing housing shown in Figure 4a was selected as the measurement object. It can be seen that the object is prepared from different materials with large variations in surface reflectance. The outer material is made of an alloy with a rough surface and has a low surface reflectivity, while the outer and inner rings in the center are made of a metal with a smooth surface and have a high reflectivity, and intensity saturation can be clearly observed in this region. Figure 4b corresponds to the phase-shifting image at the selected camera exposure time = 30 ms, and (c) shows the parcel phase map obtained by solving the calculation. (d)–(f) show the projected complementary Gray code image, the phase order, and the absolute phase map obtained by the final solution.

In order to further verify the effectiveness of the complementary Gray code phase unfolding method based on order sharing, three different phase unfolding methods were used in the experiments, namely multi-frequency unwrapping, the complementary Gray code method, and the order sharing method. And the phase solution effect under different camera exposure times was observed. The specific settings were as follows:

(1) Multi-frequency step-by-step phase unfolding: Using the low-frequency streak image information collected under the current exposure time, the parcel phase was unfolded to obtain the absolute phase.

(2) Complementary Gray code phase unfolding: Using the complementary Gray code image information collected under the current exposure time, the parcel phase was unfolded to obtain the absolute phase.

(3) Complementary Gray code phase expansion based on order sharing: The complementary Gray code image information collected under the camera’s exposure time = 30 ms with a good exposure effect was used to expand the parcel phase and obtain the absolute phase.

**Figure 5:**
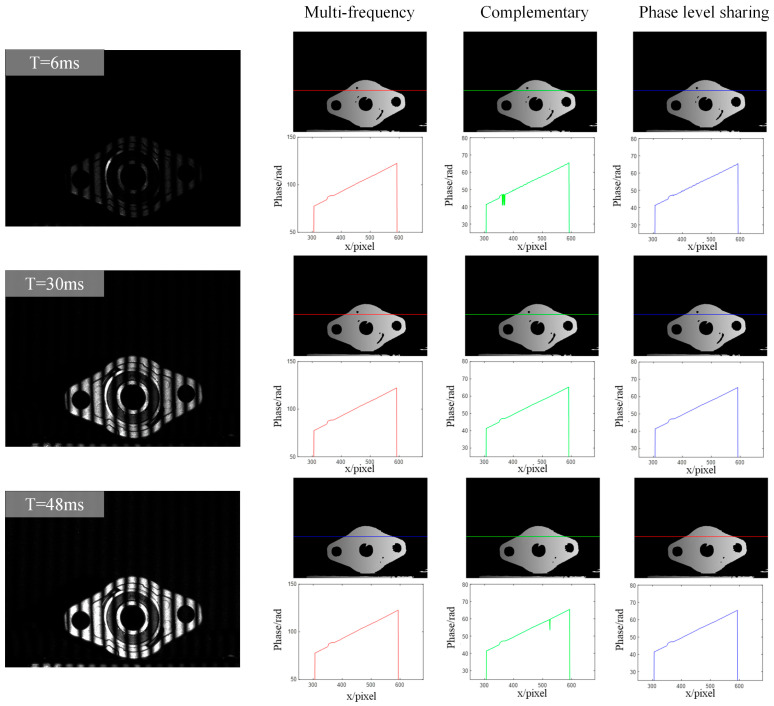
Comparison of phase solving results under different methods.

The phase resolution results corresponding to the different methods at three exposure times, short, medium, and long, are shown in Figure 5. On the left side is the acquired image of the high-frequency streak projection, and on the right side is the absolute phase map and the line graph plotted along the horizontal axis by selecting a certain row of phase values. It can be seen that with a short exposure time of 6 ms, the acquired image is overall darker and basically free of intensity saturation. And as the exposure time is prolonged, the overexposure phenomenon on the surface of the measurement object becomes more and more obvious, mainly appearing in the middle of the workpiece’s inner and outer axial ring part, with a strong reflection phenomenon. In terms of the absolute phase solution, both the step-by-step expansion and the complementary Gray code expansion based on phase order sharing can successfully expand the parcel phase in the low exposure case, while the absolute phase solved by the complementary Gray code method has a phase mutation, which indicates that there is an error in this part of the step-by-step expansion, since the exposure time is too short, which results in the black-and-white edge transition segment in the acquired Gray code projection image during binarization. In the case of medium exposure time, all three methods can correctly solve the absolute phase. It can be seen that intensity saturation occurs in many regions on the surface of the object in the case of long exposure time, and the phase solving effect of the complementary Gray code method based on phase order sharing is still better than that of the traditional complementary Gray code method, which proves that the phase order sharing method reduces the projected image and proves the selection of a group of Gray codes with appropriate exposure for decoding. The effect will also be better than a group with too weak or too strong exposure.

The phase level sharing method based on complementary Golay code refers to using the same set of Golay code patterns for phase unwrapping of phase-shift images captured under different exposure times, thereby reducing the number of projection images in HDR scenes. At the same time, by comparing a line of phase change line graphs drawn under different exposures of the same method, it can also be found that the absolute phase of the solution has a jitter phenomenon when the exposure time is shorter, which is affected by the noise, indicating that even if overexposure phenomenon does not occur when the overall grey value of the image is low, but the phase-shifting image stripes are not obvious in the contrast of the acquisition, which will also affect the accuracy of the final calculation of the absolute phase.

### 3.3. Experiments on 3D Measurement of Standard Workpieces

**Figure 6:**
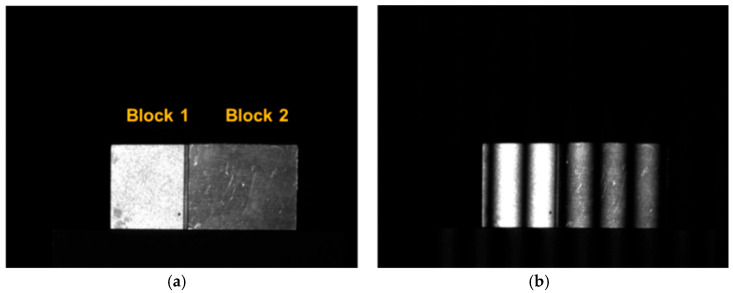
Measurement of the object’s metal mass. (**a**) The setup of gauge 1 and gauge 2; (**b**) the reflection of gauge 1 and gauge 2.

The adaptive exposure fusion method determines the optimal number of exposures and exposure time required in HDR scenes through clustering. In the experiment comparing the measurement accuracy of different methods, two precision machined standard metal gauges with thicknesses of 2 mm and 5 mm, respectively, and different surface reflectivities were used. They were denoted as gauge 1 and gauge 2, and used as measurement objects in this section. The two gauges were superimposed and fixed using a device during the measurement, and the accuracy of the different methods was evaluated by measuring the spacing between the upper and lower planes, the thickness of gauge 2 (5 mm), and the fixation of the gauge as shown in Figure 6a. Figure 6b shows the reflections on the surfaces of the two gauge blocks during the projection of a high-frequency sinusoidal stripe image, and it can be seen that there is an overexposure phenomenon for gauge block 1 while the surface of gauge block 2 is normal for the projected image at this exposure time. This experiment simulated a high-dynamic-range scenario in real industrial inspection by setting up measurement objects with different reflectivities.

The main methods used for this section were as follows:

(1) Using the phase-shifting method to calculate the wrapped phase at a single exposure time and the step-by-step unfolding method to obtain the absolute phase, as a control group under no exposure fusion, using 4 frequencies and 6 steps for a total of 24 images; this method is denoted as SE (single exposure).
(2) Using eight groups of equal-step exposure times (6 ms, 12 ms, 18 ms, …, 48 ms) and multiple-exposure fusion to obtain phase-shifting images and using multi-frequency step-by-step unfolding to obtain the absolute phase belonging to a common structured-light camera on the market to measure the high dynamic range of the processing method, with 24 images per exposure group; this method is denoted as ME (multi-exposure).
(3) Using two, four, and eight groups of equal-step exposure time and multiple-exposure fusion to obtain phase-shifting images and using complementary Gray code expansion to obtain the absolute phase, with a total of five projected Gray code images, one each for high-intensity uniform grey-scale images and when not projecting images, and six images for each group of phase-shifting images; this method is denoted as GC_i (Gray code, i is the corresponding number of projection groups).
(4) The method proposed in this section, which determines the number of exposure groups and the corresponding time by using adaptive exposure fusion method and solves the phase values by the complementary Gray code method based on phase order sharing; there are five projected Gray code images in total, one high uniform grey scale image and one low uniform grey scale image are collected with and without a projected image (surface reflection model is calculated for pre-projection), and there are six phase-shifting images in each group; this method is denoted as Ours.

**Figure 7:**
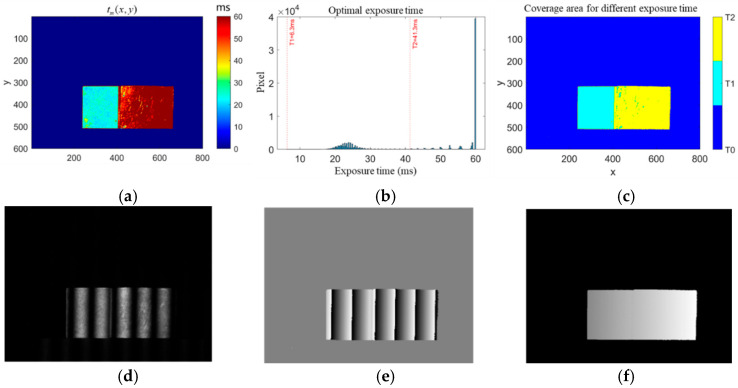
Adaptive exposure fusion method used to calculate phase results. (**a**) The ideal exposure time distribution map tm(x, y) on the object surface calculated pixel by pixel after the establishment of the surface reflectance model; (**b**) the optimal number of exposure groups is determined to be 2 after the calculation of the adaptive exposure time method; (**c**) 2 groups of exposure time adapted to different regions of the object surface; (**d**) the new phase-shifting image obtained after fusion; (**e**) the wrapped phase obtained by the phase-shifting method for solving the phase; (**f**) the absolute phase map unfolded by the complementary Gray code method using the phase order sharing method.

Figure 7 shows the process of measuring the volume blocks using the adaptive exposure fusion method proposed in this section. Figure 7a represents the ideal exposure time distribution map $t_{m}$(x, y) on the object surface calculated pixel by pixel after the establishment of the surface reflectance model, and it can be seen that the results can be roughly divided into two intervals through the distribution. Figure 7b shows that the optimal number of exposure groups is determined to be two after the calculation of the adaptive exposure time method, with one group of exposure time being 6.3ms and one group of exposure time being 41.3 ms. Figure 7c represents the two groups of exposure time adapted to different regions of the object surface, which basically divides the object into two regions, corresponding to the different surface reflectivities of the two volume blocks; Figure 7d–f show the new phase-shifting image obtained after fusion, the wrapped phase obtained by the phase-shifting method for solving the phase, and the absolute phase map unfolded by the complementary Gray code method using the phase order sharing method, respectively.

**Figure 8:**
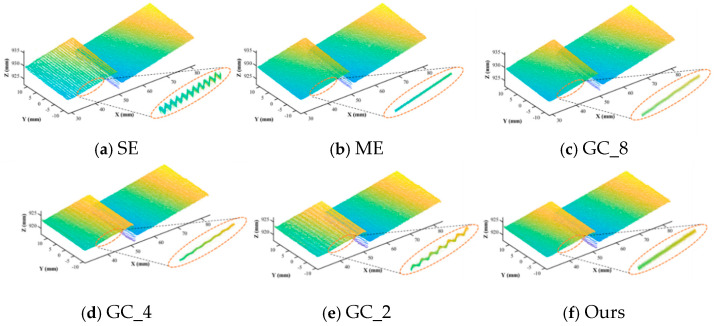
Surface point cloud results of standard parts reconstructed by different methods.

The point cloud reconstruction results of the volume block surface for different methods are shown in Figure 8 and Table 1. When only one set of exposure times (SE method) was used, the point cloud reconstruction details of the volume block 2 surface were enlarged, and it was found that the point cloud was corrugated rather than smooth planes, whereas the surface of volume block 1 was smoother in comparison. This is due to the fact that the acquired image of the volume block 1 surface at this camera exposure time is not overexposed, while the volume block 2 surface is overexposed, which leads to a phase resolution error and further increases the error of the actual point cloud, whereas, when the fusion of the images acquired with eight groups of fixed-step exposure time is used and then the multi-frequency stepwise expansion (ME) or complementary Gray code (GC_8) method is used to calculate the point cloud, the point clouds of the surfaces of volume blocks 1 and 2 are all smoother than a single group. The data are smoother and more in line with the actual data than a single group. However, as the number of exposure groups decreases to the point where only two groups of exposure time fusion are used, the computed surface of volume block 2 still appears to be rippled. This is due to the fact that even though multiple-exposure fusion is used, the choice of exposure time can only be determined empirically; the determined exposure time is not optimal, and the phase-shifting image obtained after fusion still has the problem of intensity saturation. Figure 8f shows the point cloud calculated by the adaptive exposure fusion method proposed in this paper, which uses two sets of exposure time images for fusion as in Figure 8e, but its exposure time is calculated adaptively according to the surface reflection model, and the surface of the reconstructed point cloud is also flatter.

In order to quantify the measurement accuracy of the method proposed in this section, the height of gauge block 2 was selected as the measurement term, and the thickness was measured by taking the points in the fixed regions of the upper and lower surfaces for plane fitting and finding the mean value of the distances from the points in the upper surface region to the fitted plane on the lower surface, as well as the mean value of the distances from the points in the lower surface region to the fitted plane on the upper surface as the measurement value H, and the standard deviation of the distances std was used as a measure of the distribution of the error. The above operations can be performed using the point cloud processing software CloudCompare 2.11.0.

**Figure 9:**
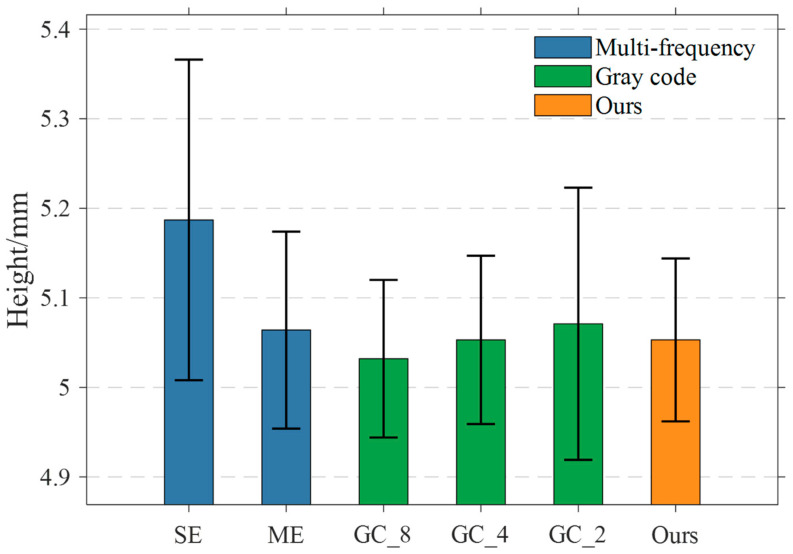
Block thickness measurement results for different methods.

The measurement results are recorded as shown in Figure 9. It can be seen that, without the use of multi-exposure fusion, the single-exposure method SE measurement error is the largest, and the data fluctuation is large. The error is the smallest with the use of eight groups of equal-step exposure time fusion for the phase-shifting method and the phase order sharing complementary Gray code for phase resolution of GC_8. For this paper’s proposed method, the use of two groups of exposure time under the phase-shifting image for the fusion, the measured value is 5.053 mm, the error is 0.053 mm, and the standard deviation is 0.091 mm. The error and standard deviation are reduced by 25.4% and 40.1%, respectively, compared with the GC_2 method using two groups of images, and the measurement accuracy is basically the same as that of GC_4 using four groups of images and ME using eight groups of images. However, the method in this paper reduces the number of images used from 31 to 20 compared to GC_4, which is a reduction of about 35%, and reduces the number of images by about 79% compared to the ME method, which does not use phase order sharing, which verifies that the proposed method in this paper is able to determine the optimal number of exposure groups and time efficiently and has the advantages of self-adaptation, fewer projected images, and higher accuracy compared to the traditional method.

## 4. Conclusions

In this paper, an adaptive exposure time selection method is proposed to calculate the optimal number of exposures and exposure times by using an improved clustering method to divide regions with different reflection degrees. At the same time, the phase order sharing strategy is adopted in the phase unwrapping stage, and the same set of complementary Gray code patterns is used to calculate the phase orders under different exposure times. The experimental results verify the effectiveness of the phase order sharing method in multiple image exposure fusion, and the comparison experiments show that the measurement error of the method described in this paper was reduced by 25.4% under almost the same exposure times.

It is inevitable that there are still some limitations. The current calibration strategy is to adopt the pixel-by-pixel polynomial equation method. Although this method has high accuracy, the calibration parameters obtained are only applicable to 3D measurements within the calibration area and are not suitable for measurements outside the calibration area. In industrial production, the size range of workpieces that need to be inspected is relatively large. Subsequently, targeted improvements can be made by using calibration strategies that can measure the entire field. In addition, deep learning [52,53] and transfer learning algorithms [54,55,56] will be applied for HDR structured light 3D reconstruction.

## Figures and Tables

**Table 1:** The comparison of measurement accuracy among different methods. The true value of the gauge block height is 5.000 mm.

Method	Projected Images N_total_	Total Exposure Time T_total_/ms	Result H_i_/mm	Standard Deviation std/mm
SE	24	240	5.187	0.179
ME	192	5184	5.064	0.110
GC_8	55	1380	5.032	0.088
GC_4	31	660	5.053	0.094
GC_2	19	336	5.071	0.152
**Ours**	**20**	**381.6**	**5.053**	**0.091**

## Data Availability

Dataset available on request from the authors.
